# Cerebral microbleeds in idiopathic normal pressure hydrocephalus

**DOI:** 10.1186/s12987-016-0028-z

**Published:** 2016-02-10

**Authors:** Elias Johansson, Khalid Ambarki, Richard Birgander, Nazila Bahrami, Anders Eklund, Jan Malm

**Affiliations:** 1 Department of Pharmacology and Clinical Neuroscience, Norrlands Universitetssjukhus, Umeå University, S-901 85 Umeå, Sweden; 2 Department of Radiation Sciences, Umeå University, Umeå, Sweden; 3 Centre for Biomedical Engineering and Physics, Umeå University, Umeå, Sweden

**Keywords:** Idiopathic normal pressure hydrocephalus, Magnetic resonance imaging, Cerebral microbleeds

## Abstract

**Background:**

A vascular disease could be involved in pathophysiology of normal pressure hydrocephalus (INPH). If so, there should be an association between INPH and cerebral microbleeds (CMB). This study aims to analyze if CMB are associated with INPH.

**Methods:**

In this case-control study we included 14 patients with INPH (mean age 76 years, 60 % female) and 41 healthy controls (HeCo; mean age 71 years, 60 % female). All were investigated with magnetic resonance imaging (MRI) using a T2*-sequence. The MRI exams were reviewed by two neuroradiologists for the presence of CMBs; the prevalence of findings of two or more CMBs was compared between INPH group and control group. After investigation, INPH patients underwent shunt surgery.

**Results:**

Two or more CMB were detected more frequently in the INPH group compared to HeCo (n = 6, 43 % vs. n = 4, 10 %; *p* = 0.01). Among the participants where MRI revealed CMB, the number of CMB was higher among the INPH patients than the HeCo (median 8; IQR 2-34 vs. median 1; IQR 1–2; *p* = 0.005).

**Conclusions:**

This study supports a vascular component to the pathophysiology of INPH.

## Background

Idiopathic normal pressure hydrocephalus is a syndrome featuring communicating hydrocephalus in combination with a symptom triad of balance- and gait-disturbance, cognitive decline and urinary incontinence. In Sweden, the prevalence in the 70–79 year age group is 0.2 %, rising to nearly 6 % in individuals over 80 years [1]. The pathophysiology is probably multi-factorial [2]. As INPH patients improve with cerebrospinal fluid (CSF) drainage, a CSF dynamic disturbance is usually considered the main etiological component. In recent years, small vessel disease has emerged as an alternative mechanism to explain part of the pathophysiological process in that vascular risk factors, manifest vascular disease, and white matter hyperintensities have all been associated with INPH [3].

Cerebral microbleeds are iron deposits in the brain that are associated pathologically with hemosiderin-laden macrophages [4]. Mainly located around small vessels, and revealed in iron-sensitive MRI sequences, CMB are associated with moderate cognitive decline, several causes of dementia and several vascular risk factors including age, hypertension, lacunar infarctions, white matter lesions and diabetes [4–9]. However, the association between CMB and INPH has not been studied. The vascular hypothesis that part of the INPH syndrome could be explained by small vessel disease, would be strengthened if there is an association between INPH and CMB. The aim of this study was to analyze if CMB are associated with INPH.

## Methods

This was a retrospective case-control study. From a consecutive cohort of patients aged >40 years that underwent evaluation for ventriculomegaly, we included all (n = 14) patients that: (1) were classified as probable (n = 9) or possible (n = 5) INPH according to guidelines [10] (2) underwent shunt surgery and (3) were examined with MRI including a T2*-sequence preoperatively. The included patients were evaluated between January 2008 and March 2009. Mean Evans index was 0.40 (range 0.36–0.49). Mean symptom duration was 3.2 years (range 1–7). All INPH patients had gait disturbance (100 %), 13 (93 %) had incontinence and six (43 %) a mild cognitive impairment (defined as Mini Mental Examination score of 22–26). Cognitive status and number of vascular risk factors were not exclusion criteria for the INPH-patients as they were for the controls. All patients were treated with a ventriculo-peritoneal shunt. Post-operative improvement was defined as reduced need of walking aids and/or walking speed increased by >15 %, assessed by comparison of pre-operative and post-operative standardized exams of gait and walking speed performed by experienced physiotherapists at our neurology ward. The mean delay between surgery and post-operative assessment was 17 weeks (range 13–24 weeks). Post-operative follow-up was available in 13 patients. Of these 13 patients, ten (77 %) improved, one (8 %) did not improve despite that the shunt was patent. Two (15 %) patients died from perioperative complications within one month of the surgery.

Forty-one healthy controls (HeCo), who considered themselves healthy, were recruited between September 2007 and August 2008 through an advertisement in the local newspaper. HeCo were eligible if they did not have any psychiatric or neurologic disorder or signs of manifest atherosclerotic disease (such as stroke or myocardial infarction). Mini-mental state estimation ≥28 points was required, without any contraindication for MRI examination. Not more than two vascular risk factors out of smoking, hypertension and hyperlipidemia were accepted, potential controls with three vascular risk factors were not enrolled in the study.

All subjects (patients and HeCo) were examined with MRI using a hydrocephalus protocol with a Philips Achieva 3 Tesla machine and an 8-channel head coil. Beside standard T1, T2 and FLAIR sequences, the CMB were assessed using a T2*-weighted sequence (axial T2 FFE, 5 mm slice thickness and 1 mm intersection gap (FOV = 230 × 230 mm, matrix acquisition 512x512, TE/TR = 833 ms/16 ms). The occurrences of CMB were independently evaluated by two senior neuroradiologists (NB, RB) using the T2* images. The other MRI data were used to rule out CMB-mimics and disagreements were resolved by consensus decision. The number of microbleeds was assessed using the Microbleed Anatomic Rating Scale (MARS) [11].

CMB were analyzed using median, intra-quartile range (IQR). Mann–Whitney test was used to compared INPH and controls. Cohen’s Kappa values were calculated for inter-observer agreements. Where appropriate, we used 95 % CI, χ^2^-2-test and *t* test. A *p* value of <0.05 was pre-selected as the threshold for statistical significance. IBM SPSS v20.0 software was used for all statistical analyses.

The study was approved by the regional ethical review board at Umeå University.

## Results

This study included 55 subjects; 41 healthy controls and 14 INPH patients. See Table 1 for baseline comparisons. Altogether, 249 CMBs were observed in all subjects. Among patients with INPH, the median number of CMB was 0.5 (IQR 0–8), and among HeCo the median number of CMB was 0 (IQR 0–1; *p* = 0.035). When only subjects with CMB were taken into account, the median number CMB was eight (IQR 2–34) in INPH and one (IQR 1–2) among the controls (*p* = 0.005).

**Table 1:** Baseline comparison between patients with idiopathic normal pressure hydrocephalus and healthy controls

	INPH (n = 14)	Healthy controls (n = 41)	*P* value
Age mean (SD)	76.4 (5.1)	70.5 (5.4)	0.001*
Women n (%)	6 (43)	18 (44)	1.0^a^
Previous MI n (%)	0 (0)	0 (0)	NA
Previous stroke n (%)	0 (0)	0 (0)	NA
Current smoking n (%)	0 (0)	2 (5)	1.0^a^
Hypertension^b^ n (%)	12 (86)	28 (68)	0.30^a^
Treated hyperlipidemia n (%)	0 (0)	6 (15)	0.18^a^
0–2 risk factors^c^	14 (100)	41 (100)	NA
3 risk factors^c^	0 (0)	0 (0)	
Anti-platelet or anti-coagulant medication	4 (29)	0 (0)	0.003^a^
MMT mean (range)	26.6 (22–30)	29.3 (28–30)	0.002*

*INPH* idiopathic normal pressure hydrocephalus, *MI* myocardial infarction, *MMT* mini mental test, *SD* standard deviation

* 2-sided t-test

^a^2-sided χ^2^-test

^b^Defined as either blood pressure > 140/90 or current use of blood pressure lowering medication

^c^Refers to current smoking, hypertension and treated hyperlipidemia

The frequency of ≥2 CMB was 43 % in INPH and 10 % in HeCo (Table 2; *p* = 0.01). However, frequency of ≥1 CMB did not differ between the two groups significantly, as it was 50 and 29 % in the INPH group and control group, respectively (p = 0.20). The distribution of CMB is presented in Table 3.

**Table 2:** Number of participants with cerebral microbleeds among idiopathic normal pressure hydrocephalus patients and healthy controls

	INPH	Healthy controls
Number of subjects	14	41
0 CMB n (%)	7 (50)	29 (71)
1 CMB n (%)	1 (7)	8 (20)
≥2 CMB n (%)	6 (43)	4 (10)
≥5 CMB n (%)	4 (29)	0 (0)

**Table 3:** Location of the cerebral microbleeds among the patients with idiopathic normal pressure hydrocephalus and healthy controls

	INPH	Healthy controls
Number of subjects with a CMB	7	12
Infratentorial, n (%)	4 (57)	2 (17)
Subcortical (“deep”), n (%)	5 (71)	6 (50)
Lobar, n (%)	4 (57)	4 (57)

A concerning finding was that the two INPH patients that died within 30 days of shunt placement surgery were the two patients with the highest number of CMB (34 and 174 CMB) in the study. The patient with 174 CMB suffered an intracerebral hemorrhage and the other patient had a sepsis with intrathecal bacteremia. Agreement between the observers for the presence of ≥2 CMB was 96 % (53/55) with a kappa value of 0.89 (95 % CI 0.73–1.0), agreement between the observers for the presence/absence of any CMB was 98 % (54/55) with a kappa value of 0.96 (95 % CI 0.88–1.0).

## Discussion

This was a preliminary study, which revealed that the prevalence of CMBs was higher in patients with INPH than in healthy controls. Two serious adverse events occurred after shunt surgery, and these two patients had extensive CMB. There is no obvious causative link between CMB and the features of INPH such as large ventricles and periventricular edema [10]. The association is likely due to the fact that both CMB and INPH are associated with small vessel disease and vascular risk factors [4–7, 12]. Hence CMB are more common in patients with INPH. A similar association with vascular disease has also been proposed for CMB and dementia [9], and for CMB and increased risk of cerebral hemorrhage in patients treated with intravenous thrombolysis [13].

Our estimation of the prevalence of any CMB (50 %) was similar to that of patients with ischemic stroke (49 %) and other vascular disorders such as vascular dementia (59 %) and vascular Parkinsonism (56 %); whereas similar non-vascular degenerative disorders have lower prevalence: Non-vascular dementia 21 % and idiopathic Parkinsons disease 18 % [9, 14, 15]. Thus, our findings contribute to a better understanding of the etiology of INPH and support the suggested association between INPH, small vessel disease and vascular risk factors [6, 7]. Combined, these findings suggest that INPH is a combination of vascular disease and cerebrospinal fluid dynamic disorder. However, more detailed studies of the association between INPH and vascular risk factors are warranted. The two patients with the highest number of CMB died within 30 days after shunt placement. One of the causes of death (intracerebral hematoma) could reasonably be associated with CMB. It is well known that intracerebral hemorrhage can occur after severe complication to shunt placement [16]. As this is a small study, this might very well be a chance finding.

We used ≥2 CMB as outcome in this study. We did not use the presence of any (≥1) CMB as outcome as it is reasonable to argue that one CMB can be considered a normal variant rather than pathological: Although no CMB is most common among the elderly (85 %), the presence of any one CMB is also common (9 %); whereas only 6 % has ≥2 CMB [5, 6]. Also, ≥2 CMB is required to cause a clinical effect in diseases [13, 17]. A considerable weakness of this study is the small sample size. Another weakness of the study was that the mean age of the INPH patients was higher than for HeCo because the controls were not recruited to match specific INPH patients. Since CMB prevalence increases with age, a part of the difference in CMB prevalence might have been caused by difference in age. However, in support of this study,agreement between the reviewers (kappa 0.89) was in the upper tier compared to previous studies using T2* (kappa 0.33–0.88) [4].

## Conclusion

Almost half of patients with INPH have several (≥2) CMB and several CMB seems to be more common among patients with INPH than among controls. If these preliminary data can be confirmed, it would support the hypothesis that INPH pathophysiology includes a vascular component.

## Abbreviations

CMB: cerebral microbleeds
CSF: cerebrospinal fluid
HeCo: healthy controls
INPH: idiopathic normal pressure hydrocephalus
IQR: inter quartile range
SD: standard deviation
